# Artificial intelligence: The good, the bad and the beautifiable. A patient's view

**DOI:** 10.1016/j.fhj.2024.100167

**Published:** 2024-09-19

**Authors:** Marlene Winfield

**Affiliations:** Advisor to the NHS on patient and carer policy, London, UK

**Keywords:** Patient and public involvement, Ethical artificial intelligence, Public trust, Personal data

After decades of working on health reform, I now find myself a semi-retired person managing three long-term conditions. I know more than ever what the world looks like to someone striving to keep poor health at bay – deciding when I need professional support, what will work best for me, and how to access it. At times, navigating the health and social care landscape can be at best challenging, at worst unfathomable.

I sometimes think how nice it would be to have a health buddy to walk and talk me through the maze and, ideally, to keep me out of it in the first place. I know that human health buddies are in short supply, so what about a digital friend? In the world of artificial intelligence (AI), we are told that anything is possible.

## The good

In early testing, AI has shown itself to be as good as or better than humans at some forms of diagnosis. At breakneck speed, AI's algorithms can analyse my medical history and images (eg X-rays, MRIs, ultrasounds, CT scans, etc.) to help identify and diagnose problems more accurately and quickly.^1^

Moreover, by taking account of a huge range of information about me, AI can personalise the way that I receive information. It could, for example, take account of my preferred language, reading age, health literacy level, digital literacy level and preferred formats to tailor its responses perfectly to my communication needs. It could mash up a lot of medical and social information about me, as well as my previous computer searches, to answer my present health and care questions.

In addition, AI could help me manage my long-term conditions.^2^ It increasingly drives the digital tools that help people monitor themselves – blood pressure, blood sugar, blood oxygen, movement for wellbeing checks and fitness tracking are a few examples. Based on the results, it can offer advice on what to do more of, less of, or when to get help.

The time is not far off when AI can create for each of us the equivalent of a Citymapper, a digital buddy to guide our health and care journeys.

Like all information technology though – or all friendships, for that matter – it can have its ups and downs.

## The bad

**Fig. 1:**
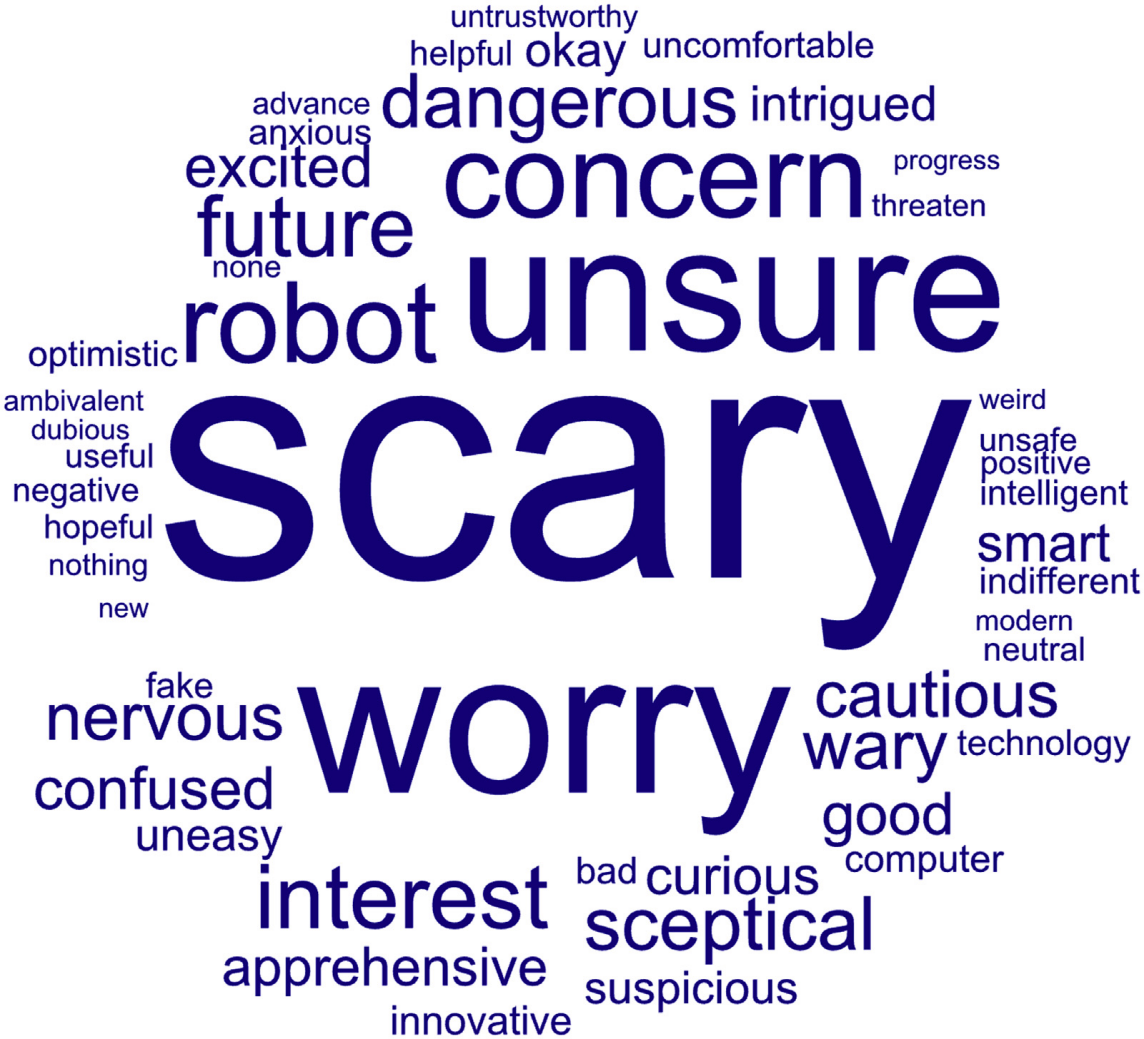
Word cloud of public sentiment towards AI by UK adults from the Centre for Data Ethics and Innovation (CDEI)’s Public Attitudes to Data and AI (PADAI) Tracker Survey. Source: Public Attitudes to Data and AI: Tracker Survey (Wave 3), UK Government. Licensed under the Open Government Licence v3.0. Available at: https://www.gov.uk/government/publications/public-attitudes-to-data-and-ai-tracker-survey-wave-3/public-attitudes-to-data-and-ai-tracker-survey-wave-3.

People's feelings about AI range from ‘exciting’ to ‘scary’ according to a recent Gov.UK public attitude tracker (Fig. 1).^3^ When asked about risks that AI poses to society, people's concerns included humans losing control over AI (34%), cyber-crime and terrorism (23%), making decisions that humans can't understand or explain (23%), and AI being biased and leading to unfair outcomes (14%). On this last point, while 43% trust AI to be unbiased, only 38% trust humans.

Interestingly, younger people, aged 18–34, were the most likely to be concerned that AI will negatively impact people's mental health and wellbeing (16%) and that biased AI will lead to unfair outcomes (16%). Given that this age group is probably the most digitally literate, how worried should I be?

The Health Foundation points out that, in healthcare, bias leading to unequal treatment could be life-threatening: ‘Without proper safeguards, AI could cause or magnify serious harms to individuals, organisations or communities, especially where it is used in patient care. We have already seen this play out – for example, in the case of a risk-prediction algorithm that failed to recommend medical treatment for Black patients.’^4^^,^^5^

To these concerns, I would add two more. I must confess that, although I like the convenience of online prescription ordering, hospital portals and access to my medical records, I would be sorry never to have the pleasure of some human contact from the NHS. I only ever need to contact my hospitals in extremis, but even then it is becoming increasingly impossible to speak to a human being. Happily, my GP surgery has gone the opposite way, improving its telephone services wonderfully. But friends complain about new automated telephone triage systems in their surgeries that frustrate and defeat them.

I would also wonder what was happening to my most personal data of all – my health data. Where was it going, could it fall into the wrong hands and compromise me in unforeseen ways, clinically or financially? In my past life at the Department of Health, I remember how some people worried that their mental or sexual health history would influence the way that they were treated for other things in the NHS. They also worried that employers or mortgage lenders would get access to information they had not given them.

For me, then, what would it take for the exciting to prevail over the scary?

## The beautifiable

The first thing to say is that transparency is king. As the National Data Guardian has noted: ‘Public trust can only be earned through a commitment to honesty and transparency. There must be no surprises for people about how their private information is being used.’^6^

As the use of AI grows, there need to be deliberate efforts to help us understand how it works and the pros and cons, so that we can do our own risk assessments, decide what we are prepared to trade for what return.

If used wisely, AI has the potential for making services feel more personal and continuous, compensating for limited resources. For long-term conditions, this could be achieved by giving care at times face to face but filling the gaps with remote monitoring. My GP surgery is planning for the future. It has beefed up its back office to be very responsive and is investigating how best to use a combination of people and technology, including AI, to support self-care in new ways.

The Health Foundation has suggested sensible ‘beautifications’ if the full benefits of AI are to be realised.^7^ They include meaningful public and staff engagement, data and digital infrastructure that are fit for purpose, high-quality testing and evaluation, clear and consistent regulation, and the right workforce skills and capability. They advise that the existing ethics frameworks and guidance are insufficient and that a renewed approach is needed to ensure that AI works for all.

Law firm Simmons and Simmons has raised legal issues that require new thinking about safeguards.^8^ They advise that ways need to be found to ensure that my consent, where required, can be as informed as possible. That means being open about the known risks of AI and giving me a certain amount of choice over to what extent I use digital tools and share my personal data. Consent and opt-outs are issues that have been grappled with for a long time in healthcare without real resolution.

There needs to be an effective regulatory framework that is updated as AI evolves. When things go wrong, there should be a workable system for deciding who is responsible – is it me, my care professionals, the digital tools, or some combination of the three? That requires a shared understanding of what is expected of all parties, aided by clear instructions. Given the recent well-known failures to compensate people harmed by faulty blood products and faulty post office IT systems, I will need to be assured that the safety net for AI-induced harms will deliver explanations and redress in a timely manner.

At a time of increasing cyber-attacks on vital systems including healthcare, cybersecurity should be given a visibly high priority in the government's security activities. Great care must be taken to eliminate, as far as possible, bias and discrimination in AI decision-making and to help me understand the basis on which decisions are made about me. The old garbage in / garbage out adage should be taken seriously.

Too often in recent times we have shown ourselves to be under-prepared for problems like pandemics, droughts, floods, energy supply issues and IT outages. I would like to feel confident that there is sufficient forethought and action to prepare for the impacts – good and bad – that our increasing dependence on AI will have on our society and on health and social care delivery.

## Conclusion

I want AI to help transform the way I manage my own health and care and to help make the NHS and social care sustainable. It is early days in AI's development. Rather like vaccinations and antibiotics, organ transplants and X-rays in their time, there are many unknowns that we must get to grips with. For most of us, our highest priority for healthcare is wellness. We are prepared to make trade-offs based on what we understand to be the risks and benefits. We saw how these trade-offs worked in the early days of internet banking and online shopping.

The best way to gain and keep my trust is to be open about the potential problems, as far as we can judge them, and clear about what measures are being put in place to manage them. I also want to feel that I have a realistic amount of control over how AI is allowed to impact my care. If all that can be done convincingly, I am prepared to welcome my digital health buddy with cautiously open arms.

## Funding

This research did not receive any specific grant from funding agencies in the public, commercial, or not-for-profit sectors.

## CRediT authorship contribution statement

**Marlene Winfield:** Writing – original draft.

## Declaration of competing interest

The authors declare that they have no known competing financial interests or personal relationships that could have appeared to influence the work reported in this paper.
